# Supplementary material for: Isolation of Extracellular Vesicles from Human Follicular Fluid: Size-Exclusion Chromatography versus Ultracentrifugation
Source: Biomolecules. 2023 Feb 2;13(2):278. doi: 10.3390/biom13020278 (PMC9953485; doi:10.3390/biom13020278)
Supplement: Supplementary file 1 [file biomolecules-13-00278-s001.zip › biomolecules-2141883-supplementary.pdf]

Supplementary material

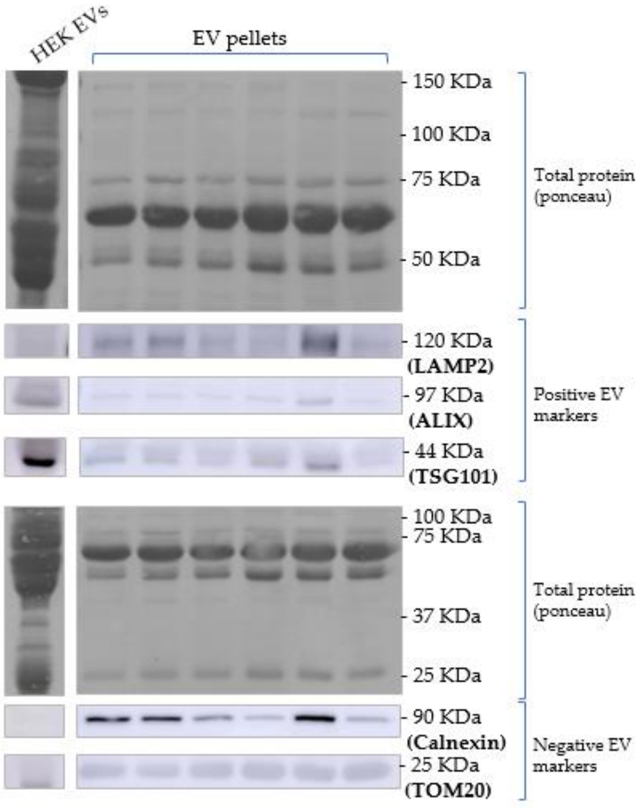

Figure S1 Representative image of western blot membrane of UC pellets showing positive and negative EV markers and the respective ponceau staining. A control lane was added to depict the positive and negative markers as well as the total protein pattern of EVs isolated from HEK cells.

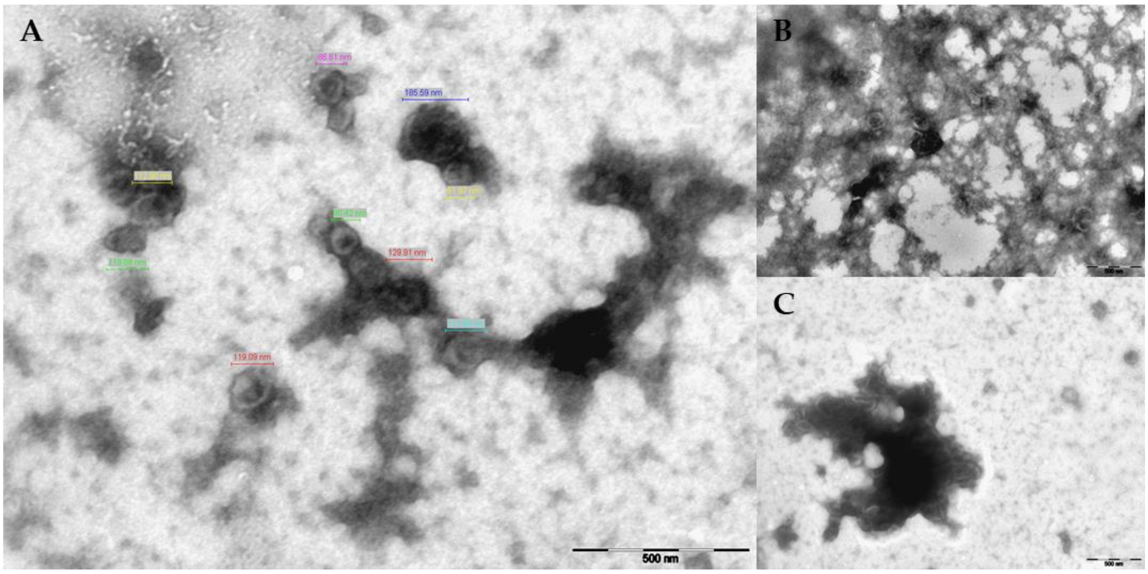

Figure S2 Representative images of EVs isolated through UC obtained by transmission electron microscopy (TEM). (A) TEM image exhibiting diameters measurement showing that the EV particles size range from 80.42 nm to 185.59 nm. (B) Representative TEM image of the protein mesh contamination; (C) Representative TEM image of large EV aggregates.

Table S3 Total protein of 30 SEC fractions of FF from 4 independent biological samples quantified by microBCA.

	FF1 (mg/mL)	FF2 (mg/mL)	FF3 (mg/mL)	FF4 (mg/mL)
**F7**	0	0	0	0
**F8**	0	0	0.004717	0
**F9**	0.001325	0.015428	0.050468	0.007697
**F10**	0.019986	0.045011	0.084156	0.034759
**F11**	0.033007	0.055438	0.09622	0.046756
**F12**	0.060682	0.092524	0.158133	0.054396
**F13**	0.181768	0.301958	0.515494	0.116247
**F14**	0.856964	0.926183	1.447908	0.358885
**F15**	1.872074	2.219889	2.986864	0.892581
**F16**	3.769814	4.581325	5.194778	2.50209
**F17**	6.031287	5.892999	7.675836	4.22907
**F18**	8.614322	8.308743	10.94218	6.85451
**F19**	9.289074	8.308743	13.11026	8.75903
**F20**	10.31175	7.836918	11.70471	8.76979
**F21**	10.47516	7.596287	10.66335	10.60437
**F22**	9.810953	6.728129	8.563554	9.80275
**F23**	8.804096	4.916321	9.126914	9.51761
**F24**	6.700768	4.175556	5.024063	8.18337
**F25**	5.377621	3.080922	5.889019	5.893985
**F26**	4.10719	1.943823	2.986864	4.245015
**F27**	2.794586	1.330451	1.999887	3.462225
**F28**	1.808816	0.537785	1.186145	2.273245
**F29**	1.102435	0.3349	0.207379	1.428585
**F30**	0.643814	0	0.275665	0.941695

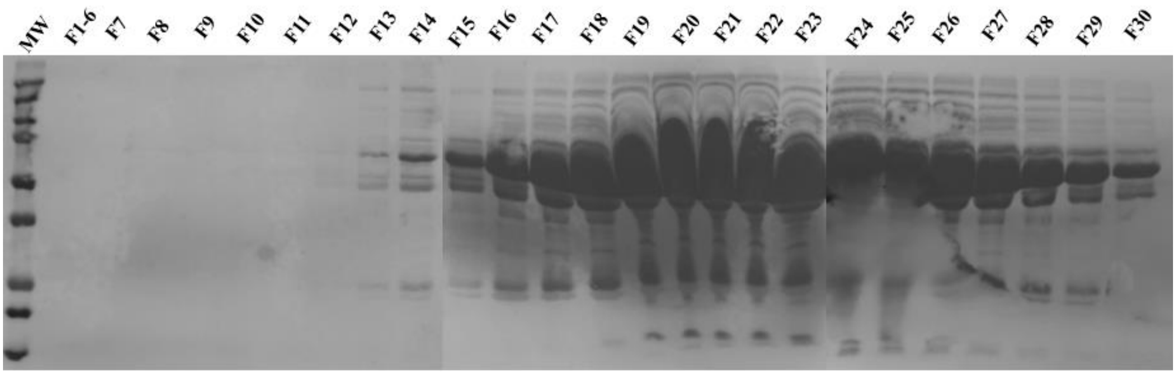

Figure S4 Representative ponceau staining of the 30 SEC eluted fractions.

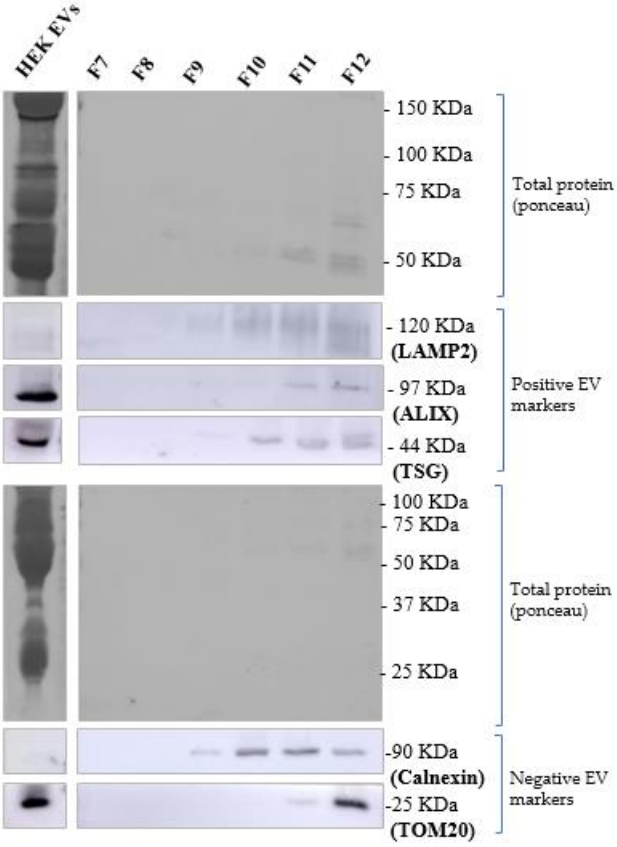

Figure S5 Representative image of western blot membrane of SEC fractions 7 to 12 showing positive and negative EV markers and the respective ponceau staining. A control lane was added to depict the positive and negative markers as well as the total protein pattern of EVs isolated from HEK cells.

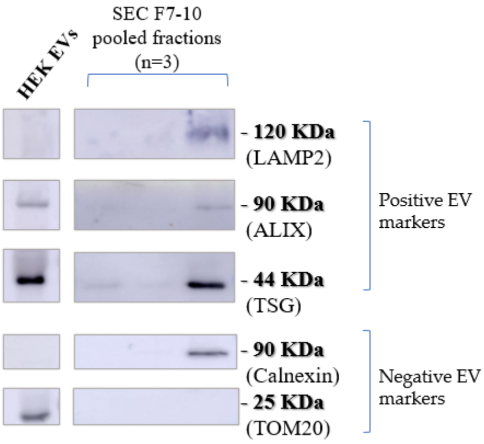

Figure S6 Representative image of western blot membrane of EV-enriched SEC fractions showing positive and negative EV markers. A control lane was added to depict the positive and negative markers of EVs isolated from HEK cells.
